# Infection prevention control and organisational patient safety culture within the context of isolation: study protocol

**DOI:** 10.1186/s12913-019-4126-x

**Published:** 2019-05-08

**Authors:** John Gammon, Julian Hunt, Sharon Williams, Sharon Daniel, Sue Rees, Sian Matthewson

**Affiliations:** 1 College of Human and Health Sciences, Swansea University, Singleton Park, Swansea, SA2 8PP Wales, UK; 2 Infection Prevention and Control, Hywel Dda University Health Board, Carmarthen, Wales, UK

**Keywords:** Isolation, Infection prevention control, Healthcare associated infection, Patient safety culture, Cultural change, Implementation theory, Qualitative research, Manchester patient safety framework, MaPSaF

## Abstract

**Background:**

Healthcare associated infection (HCAI) is a major cause of morbidity and mortality. In recent years, there have been high profile successes in infection prevention control (IPC), such as the dramatic reductions in methicillin-resistant *Staphylococcus aureus* (MRSA) bloodstream infections (which is viewed as one proxy indicator of overall harm) and *Clostridium difficile* in the UK. Nevertheless, HCAI remains a costly burden to health services, a source of concern to patients and the public and at present, is receiving priority from policy makers as it contributes to the global threat of antimicrobial resistance.

**Methods:**

The study involves qualitative case studies within isolation settings at two National Health Service (NHS) district general hospitals (DGHs) in Wales, in the UK. The 18-month study incorporates Manchester Patient Safety Framework (MaPSaF) workshops with health workers and other hospital staff, in depth interviews with patients and their relative / informal carer, health workers and hospital staff, and periods of hospital ward observation.

**Discussion:**

The present study aims to investigate the ways in which engagement of health workers with IPC strategies and principles, shape and inform organisational patient safety culture within the context of isolation in surgical, medical and admission hospital settings; and vice-versa. We want to understand the meaning of IPC ‘ownership’ for health workers; the ways in which IPC is promoted, how IPC teams operate as new challenges arise, how their effectiveness is assessed and the positioning of IPC within the broader context of organisational patient safety culture, within hospital isolation settings.

**Electronic supplementary material:**

The online version of this article (10.1186/s12913-019-4126-x) contains supplementary material, which is available to authorized users.

## Background

In recent years, the quality of care of people in institutional settings in the NHS has come under scrutiny following a number of high profile cases. HCAIs, largely preventable adverse events defined as an infection acquired as a consequence of a person’s treatment by a healthcare provider [1], are a global patient safety problem [2]. Over the past decade, literature continues to conclude that HCAIs are frequent, catastrophic and costly [3–7].

In recent times, there have been high profile successes in IPC, such as the dramatic reductions in MRSA bloodstream infections (which is viewed as one proxy indicator of overall harm) and *Clostridium difficile* in the UK [8–11]. However, HCAIs continue to occur and present a risk to patients and users of healthcare. In September 2013, the UK government published their five year strategy for tackling antimicrobial resistance [12]. In Wales, the first Welsh Government Delivery Plan specifically relating to antimicrobial resistance was produced in March, 2016 [13]. The plan included seven delivery themes. The first theme focusses on improving IPC practice. In 2014, the Welsh Government followed up the 2011 framework of actions towards the elimination of HCAIs with a Code of Practice for the prevention and control of HCAIs [14, 15]. The Code of Practice set out the minimum necessary IPC arrangements for healthcare providers in Wales. Theme one of the new Antimicrobial Resistance Delivery Plan focuses on ensuring the full implementation of this Code of Practice and compliance of other existing policies. Specific priorities address the management of patients with carbapenem resistant infections. HCAIs have thus become a major patient safety issue in the NHS.

Patient safety has been defined as ‘the avoidance, prevention and amelioration of adverse outcomes or injuries stemming from the process of healthcare’ [16], and attention has largely focused on the epidemiology and prevention of adverse events. More recently, thought has been given to understanding the shared attitudes, beliefs, values and assumptions that underlie peoples’ actions in regard to issues of safety; and of the potential importance of these shared characteristics in initiating sustained changes within patient safety [17–19]. Within the literature, these shared characteristics are often referred to as the ‘safety culture’ of an organisation [20].

### Project description

This study will look at the relationship between IPC and patient safety culture within the context of isolation, within surgical, medical and admission hospital settings. For the purposes of this research, we are interested in examining the ways in which health workers engage, or otherwise, with IPC strategies and principles, and in turn, explore what this means for organisational patient safety culture.

Our primary research question is:

- ➢ In what ways, if any, does health workers’ levels of engagement, for example, compliance with and adherence to, IPC strategies and principles, shape and inform organisational patient safety culture within isolation in surgical, medical and admission hospital settings; and vice-versa?

In view of the challenges involved in implementing ‘top-down’ IPC initiatives [21–24], contemporary thought has turned to notions that IPC should be the responsibility of all healthcare workers and the concept of frontline staff adopting ‘ownership’ is stressed in international guidelines [25–27]. For Zimmerman [28], ownership involves health workers’ identifying IPC problems within their own clinical areas, implementing solutions and drawing on the expertise of IPC practitioners, as necessary. Zimmerman studied five Canadian hospitals and identified effective communication between frontline health workers, IPC specialists and managers; encouragement of staff to share ideas and promote good practice; innovative interventions meeting localised needs and a climate of learning from mistakes thus enabling continuously improved performance; as being crucial to ownership. For Zimmerman [28], the realisation of ownership involved frontline staff receiving and responding to local metrics, while remaining constantly mindful of IPC and engaging in change.

Hospital isolation involves the physical separation of patients with infections (or suspected of infections) to interrupt the transmission of potential pathogens between other patients, staff and visitors; and has historically been used to control and prevent the spread of infectious diseases. In the UK, there has been a move towards isolation in single rooms on general wards, rather than more dedicated isolation wards [29]. Infections spread by airborne, droplet or contact categories, placing the patient in single room isolation is considered an important element of transmission based precautions (TBPs) [30–33]. TBPs are recommended when infectious agents are present or suspected, and where standard precautions alone would not prevent the spread of infectious diseases and pathogens such as MRSA, *Clostridium difficile* and Norovirus. These precautions involve patient placement, the use of personal protective equipment (PPE) (For example: gloves, gowns, masks and eye protection), hand hygiene, the decontamination of equipment and of the environment, and the appropriate management of linen and waste. The barrier of a single room is a further reminder to the healthcare worker of the necessary procedures involved in the practice of isolation [34, 35]. Even while there is much debate regarding the effectiveness of isolation precautions [36, 37], the practice is grounded on a sound theoretical rationale and is broadly accepted.

There are a number of challenges involved in implementing isolation practice and broader IPC precautions. Healthcare workers, in addition to patients and visitors, need to conform to strict protocols without compromising patient safety. Isolation or other forms of constraints have serious impact upon a patients’ health, welfare and liberty, and patients’ perspectives of isolation suggest that the imposed environment and isolation procedures, provide barriers to physical, sensory and psychosocial needs that impact adversely on the unseen burden of illness [38–43].

Healthcare can be understood as a complex adaptive system. Drawing on the work of Zimmerman [28], this qualitative study will explore the ways in which, if at all, the notion of ‘ownership’ is being played out on the ground and the ways in which this shapes and informs patient safety culture. In doing so, we will be mindful of interlinked tensions that may exist, such as: The challenge of coupling together the contributions of evidence based medicine and practice based evidence, as well as the critical role of distributed, problem focused leadership.

### Aims and objectives

This study seeks to provide new evidence on the relationship between patient safety culture and health workers’ engagement with IPC principles and strategies within isolation healthcare settings, from the perspective of people working in healthcare and from those placed in isolation.

The objectives of the study are:

- ➢ To determine the feasibility of identifying good quality and poor quality IPC strategies and principles within isolation in two DGH settings within Wales.
- ➢ To identify and examine organisational factors that promote good quality and poor quality IPC principles and strategies within isolation settings, and relate these to patient safety culture.
- ➢ Identify and understand the ways in which IPC principles and strategies shape and inform patient safety culture within isolation healthcare settings, and vice-versa.

Our research questions are:

- ➢ What does organisational patient safety culture look like within isolation healthcare settings at two DGHs, within Wales?
- ➢ In what ways does organisational patient safety culture impact on health workers’ engagement with IPC principles and strategies in isolation healthcare settings?
- ➢ In what ways do health workers in isolation healthcare settings understand the meaning of IPC ownership?
- ➢ In what ways do health workers, patients and relative / informal carers in isolation healthcare settings understand the meaning of patient safety culture?
- ➢ What do hospital staff, patients and relative / informal carers in isolation healthcare settings understand by good quality and poor quality patient safety initiatives and practices?

## Theoretical positioning

### Defining culture

In researching the culture of an organisation, a number of studies approach organisations as mini-societies [44, 45]. While there is little consensus regarding the precise meaning of organisational culture [46–48], most definitions acknowledge the ways in which the phenomenon has been symbolically constructed; positioning its generation in pervasive, normative beliefs and values, while viewing its expression in terms of patterns of behaviour [49]. For these reasons, in particular, this study draws on the writings of Schein [50], who observed organisational culture as being:

The pattern of shared basic assumptions - invented, discovered or developed by a given group as it learns to cope with its problems of external adaptation and internal integration - That has worked well enough to be valid and, therefore, to be taught to new members as the correct way to perceive, think, and feel in relation to those problems.

Cultures are thus characterised from one another by account of their considerable and varying pools of tacit knowledge - Which people belonging to those cultures understand but are not necessarily mindful of knowing [51]. In this way, culture is not simply observable in social life, it further involves the shared cognitive and symbolic context within which a society or institution can be realised.

The culture of an organisation can be understood as being layered in nature. Schein’s [50] offers a framework identifying three levels of ascending importance:

- Level 1: *Artefacts* - The most visible manifestations of culture; including rituals, rewards and ceremonies. Artefacts are especially concerned with the observable patterns of behaviour within organisations.
- Level 2: *Beliefs and Values* - Espoused beliefs and values which may be drawn on in justifying particular patterns of behaviour and which form the basis in choosing between alternative courses of action.
- Level 3: *Assumptions* - The unconscious beliefs, values and expectations held and shared by individuals; these may be signalled by artefacts that belie the espoused beliefs and values.

While there has been much theorising regarding the differing levels of culture, little work to date has captured the real and largely unspoken assumptions of culture [51–55].

### Implementation theory

A pressing problem within research in health and social sciences, has been the struggle to understand the ways in which innovations become routinely incorporated or embedded in everyday practice [56–58]. May offers a theory of implementation that realises implementation processes as interactions between ‘emergent expressions of agencies’ (the ways in which people make things happen through working with the different mechanisms shaping a complex intervention) and as ‘dynamic elements of context’: That is, the structural and cognitive resources people utilise to realise that agency [59]. May’s theoretical framework brings together four constructs:

- Capability: The capability of agents to operationalise a complex intervention depends on its workability and integration within a social system.
- Capacity: The incorporation of a complex intervention within a social system depends on agents’ capacity to cooperate and coordinate their actions.
- Potential: The translation of capacity in to collective action depends on agents’ potential to enact the complex intervention.
- Contribution: The implementation of a complex intervention depends on agents’ continuous contributions that carry forward in time and space.

The accomplishment of implementation is drawn from the social processes formed by these four constructs. Each construct has regard for the dynamic elements and objects of implementation, as well as for the potential and actual expressions of agency [59]. Thus the constructs are neither static nor linear. Rather, they are in a process of continuous interaction with each other in emergent and complex ways. Importantly, these constructs and their relationships with each other are not resistant to formalisation but form part of a social system defined as being a collection of ‘socially organised, dynamic and contingent relations’ [59].

In this research study, we will utilise elements of implementation theory in analysing processes of embedding integration (of innovations in IPC and patient safety practices) in the delivery of healthcare services within isolation settings at two DGHs within one health board in Wales. The starting position of implementation theory is an assertion that in order to understand the embedding of a practice, we must observe what people actually do in their working lives. The ways in which people express their agency (the ability to make things happen through our own actions) is through interactions with other agents, other processes and contexts. Implementation thus needs to be understood as a process rather than as a final outcome.

## Methods/Design

This study involves academics and professional IPC healthcare staff in an interdisciplinary research team collaborating in design through to dissemination.

Two phases guide our plan of investigation:

- ➢ Phase 1: The study will adopt a qualitative design incorporating MaPSaF, which will assist us in observing levels of patient safety culture maturity in isolation settings at two DGHs within Wales.
- ➢ Phase 2: Phase 1 data will be supplemented by case studies involving qualitative semi-structured interviews and periods of observation, to provide a more in-depth understanding of process, experience and outcomes, from the perspectives of healthcare workers, patients and their relative / informal carers.

Time Schedule:

- ➢ Months 1–3: Focus on project management - Gaining ethical approval, recruiting and negotiating access to research sites, and updating the study literature review. The recruiting of healthcare staff to participate in Phase 1 MaPSaF workshops will take place within this 3 month period.
- ➢ Months 4–8: Focus on the holding of and initial analysis of Phase 1 MaPSaF workshops. MaPSaF workshops will be held at four months and again at seven months, at both hospital sites. Recruitment of study participants of Phase 2 will take place within this period (Months 5–7).
- ➢ Months 8–15: Phase 2 interviews will take place. To improve efficiency of the study, the interview work will occur in parallel with periods of ward observation. Initial data analysis will take place concurrently with data collection. During this period, synthesis of Phases 1 & 2 data will commence (Months 12–15). The initial stages of study report writing will being in month 14.
- ➢ Months 16–18: Analysis and synthesis of Phases 1 & 2 data along with report writing will continue through this period. This will then be written up into a final report. A study dissemination seminar will be held at the end of the 18 month period.

### Case study site selection and recruitment strategies

Case Studies will be undertaken within isolation settings at two DGHs. The case studies will study in detail patients, staff and the environment of three wards of interest in isolation settings at each of the two hospital sites: Two wards within acute care (medical and surgical) and an admissions unit. Thus, 6 wards across isolation settings at both hospital sites. The case study sites reflect a range of organisational and system characteristics which may impact on IPC and patient safety culture.

The case study selection criteria included:

- ➢ Location Factors: Two DGHs have been selected in Wales. One hospital is in a town setting and the other in a rural setting. This will enable us to explore the relationships, experiences and differences of IPC ownership, practices and procedures, in addition to patient safety culture in isolation healthcare settings in different geographical and demographic locations.
- ➢ Organisational Factors 1: The utilisation of single room isolation standard precautions, in addition, where necessary, to TBPs, is a cornerstone of hospital IPC practice and procedure, and is taken for patients known or suspected to be infected or colonised with pathogens spread by air, droplet or contact routes. Focussing this study within isolation settings, draws on previous research and subject expertise of members of the research team. Isolation has been coupled to improvement science and thus patient safety, in order to widen the breadth of the study and to understand the significance of patient safety culture.
- ➢ Organisational Factors 2: One surgical ward, one medical ward and an admissions unit in each of the hospital sites have been selected to ensure patient safety culture and IPC within isolation settings, in a broad spectrum of NHS hospital ward settings, will be studied. Organisational factors including the size of an organisation, its functions and roles and organisational factors contributing to the ethos of patient safety and IPC can thus be studied.
- ➢ The willingness of the identified health board to become involved in the study. It is anticipated that the identified health board will become a demonstrator site for promoting excellence in IPC and patient safety culture, and in developing an open learning culture in regard to IPC ownership.

### Phase 1

Phase one of our study will involve MaPSaF workshops with healthcare staff that will be held at both DGHs. MaPSaF is designed specifically for use in the NHS and provides a view of safety culture on ten dimensions at five progressive levels of safety maturity.

The utilisation of MaPSaF allows the generation of a profile of safety culture within each hospital site in terms of areas of relative strength and challenge, which can then be used to identify focus issues for change and improvement. The effectiveness of MaPSaF in phase one will depend on the development of a shared understanding among healthcare staff of the underlying meanings of the results of the assessment, and subsequently identifying the means by which improvements in patient safety culture can be implemented and improved.

MaPSaF workshops will be facilitated by the study researchers and will held at four months and again at seven months, at both hospital sites. Each workshop will involve between 10 and 12 participants (hospital / healthcare staff) and will last around 90 min. Recruitment of participants will be facilitated by the study Local Collaborator. Research participants will be asked to consent to participate in the workshops and in completing the MaPSaF process. Participants will be advised as to the purpose of the workshop and reassured that it is not a performance management exercise, rather a process of self-reflection. MaPSaF workshops will take the form of individual evaluations, comparisons of individual evaluations and open discussion of evaluation findings. This will allow us to examine and assess levels of maturity of patient safety culture across each of the two hospital sites; as well as enabling understanding of changes stimulated by the MaPSaF process, if any.

By involving healthcare staff, we anticipate that the utilisation of MaPSaF in our study will have a range of uses, including:

- ➢ Raising awareness regarding patient safety and illustrating any differences in perception between staff.
- ➢ Stimulating discussion about the strengths and weaknesses of patient safety culture within the hospital isolation setting.
- ➢ Identifying areas for improvement.
- ➢ Evaluating patient safety interventions and innovations, and tracking changes over time.

MaPSaF Workshops - Inclusion criteria: The assessment process will require the commitment of staff and time. It will be important to ensure that regular members of staff are involved in phase one of our study as the findings are likely to point to a number of different areas in which the prevailing safety culture could be improved:

- ➢ Participants must have responsibilities within isolation settings, within the hospital, that are associated with patient care (Managers, assistant managers, allied health professionals, consultants, nurses, healthcare workers and other support staff, including cleaning and catering staff).
- ➢ Participants must have completed their preceptorship / probationary period of transition. It is felt that such experience is needed to have adequate understanding of patient safety culture within isolation hospital settings.
- ➢ Willingness to give informed consent.

Our MaPSaF workshop pack for phase one includes: A participant information sheet, MaPSaF and an informed consent form.

### Phase 2

In phase two, case studies within each of the two hospital sites will be carried out. Meetings will be held with senior managers and clinicians. An analysis of organisational IPC policies and processes, and educational / training opportunities and initiatives will be carried out. Semi structured interviews will take place with patients recently discharged from the study hospitals and their informal carers / relatives. Interviews will further be held with key staff to identify broader influences on IPC practices and principles, and with ward staff to include their perspective. Observation of the processes and practices on hospital wards in each of the two DGHs will also take place. Our aim is to build as complete a picture as possible based upon multiple perspectives relating to IPC and organisational patient safety culture.

### Study participants and recruitment strategies

Study Participants:

- ➢ Hospital patients: 4–8 weeks post-discharge from hospital isolation, together with their nominated relative / informal carers. Prospective participants will be identified by senior clinical staff and purposive sampling will ensure diversity. Hospital patients will be asked for permission to contact their relative / informal carer.
- ➢ Key staff: In positions of clinical leadership or management in the 2 hospitals sites. Responsibility for IPC and patient safety.
- ➢ Ward staff: In each of the 6 wards in the 2 hospitals sites. A series of brief daily meetings for staff on each of the potential wards will be arranged to explain the nature of the study, how they might be involved and to answer their questions. This will ensure that all potential participants will have the opportunity to directly question the researchers.

Participant Interview Numbers:

- ➢ Hospital patients: 12
- ➢ Relative / Informal Carers: 12
- ➢ Key staff: 4
- ➢ Ward staff: 12

Written information about the study aims, together with what participation involves, will be given to potential participants. Written consent will be obtained at the interview to ensure full understanding. All participants will be given assurances that their confidentiality and anonymity will be protected. This will be explained ensuring that participants have a clear understanding that immediately following transcription of the interview, no identifying information would be held about them or associated with their responses. A contact telephone number will be given to all participants should any questions not addressed at the initial contact arise. Interview topic guides for all participants were specifically developed for this study and are included here as Additional files 1, 2, 3 and 4.

### Periods of observation

Periods of observation of processes, practice and the environment will take place on 6 wards across the 2 hospital case study sites. The wards will be chosen in consultation with senior health board staff. 2 wards within acute care (medical and surgical) and an admissions unit, will be studied in each of the DGHs.

All staff and in-patients in these locations will be notified that observation will take place only within public areas of the ward. Assurances will be given that intimate care will not be directly observed as this may infringe dignity (For example: Observation will not take place in private areas such as in bathrooms, private cubicles or behind screens / curtains). Staff and patients will be informed of when researchers will be available to answer any questions they may have.

The periods of ward observation will involve approximately 48 h of observation (8 h per ward).

## Research method of analysis

Phases 1 & 2 - MaPSaF Workshops and Case Study Analysis: Discussions and interviews will be transcribed, and anonymised. Observational field notes will be written up in full and again anonymised. The data set, both MaPSaF and case studies data, will be stored electronically on a shared data base. A computer assisted qualitative data analysis software package will be utilised to manage and analyse data. Themes will be created to identify and couple data in meaningful ways [60]. Analysis will involve within-case (For example: Single hospital / ward) and across-case examination and theorisation [61], in richly describing the emerging relationship between patient safety culture and IPC.

There will be regular research team meetings to ensure consistency, while allowing developing analysis to be further explored.

### Involvement of stakeholders

The research team has extensive experience of actively involving stakeholders in research and has ready access to networks to facilitate this.

Stakeholders in this project include patients, relatives / informal carers, healthcare professionals, professional bodies, policy makers and the NHS. They will be variously involved as members of the project advisory group, members of the research team and participants. This broad level of engagement will help us to quickly disseminate our findings to a wide range of stakeholders ensuring that the new knowledge we generate will quickly get to those who can best make use of it.

The project advisory group will meet on three occasions throughout the lifetime of the project to advise and receive reports on project progress and conduct.

## Discussion

Focusing on the relationship between IPC and patient safety culture within the context of isolation is both timely and particularly interesting. The engagement of health workers with IPC and patient safety procedures and practices takes place in complex organisational environments and in circumstances where time and resources are most often stretched, and where work of one form is constantly squeezed by other demands. Nevertheless, it is imperative to understand the ways in which IPC strategies, principles and innovations are implemented and operate on the ground as new challenges arise and threats of antimicrobial resistance increases, and of the positioning of IPC within the broader context of organisational patient safety culture, within hospital isolation settings.

The strength of this study is the utilisation of a combination of different qualitative research methods: Literature search, documentary analysis, MaPSaF workshops, interviews and periods of observation. This will generate data offering new insights, enabling us to inform and support NHS organisations in regard to IPC and patient safety policies and practices, as well as patient safety culture. The selection of study interviewees is drawn from the literature and previous research experiences of the project team members. We approximate with the planned number of interviewees, data saturation will be reached.

There is strong collaboration involved in this study encompassing academics and professional healthcare staff specialising in IPC, working together in research design through to dissemination. The nature of the multidisciplinary project team representing disciplines such IPC, patient safety, improvement science, organisational culture, nursing, ethics, health policy and practice, healthcare education, qualitative methodologies and medical sociology, will minimise bias toward a specific theoretical position.

Understanding the ways in which IPC innovations are presented, implemented and engaged with by health workers and what that means for organisational patient safety culture, is essential to driving improvements in healthcare and clinical practice. Knowledge, awareness and understanding of culture and cultural change in hospital settings is crucial to promoting and delivering good quality care and patient safety within the NHS. This study offers the possibility of generating new understandings of the ways in which health workers engagement with and ownership of IPC innovations, inform and shape organisational patient safety culture within hospital isolation settings, and vice versa; that can inform future studies enabling comparisons within Wales and between Wales and other countries.

## Additional files

Additional file 1: Interview Topic Guide - Patient. (DOCX 18 kb)

Additional file 2: Interview Topic Guide - Relative / Informal Carer. (DOCX 18 kb)

Additional file 3: Interview Topic Guide - Key Staff. (DOCX 18 kb)

Additional file 4: Interview Topic Guide - Ward Staff. (DOCX 19 kb)

## Abbreviations

DGHs: District general hospitals
HCAI: Healthcare associated infection
IPC: infection prevention control
MaPSaF: Manchester Patient Safety Framework
MRSA: Methicillin-resistant *Staphylococcus aureus*
NHS: National Health Service
TBPs: Transmission based precautions

## References

[1] Department of Health. The Health Act. Code of practice for the prevention and control of healthcare associated infections. London: Department of Health; 2006.

[2] Harbarth S, Sax H, Gastmeier P (2003). The preventable proportion of nosocomial infections: an overview of published reports. J Hosp Infect..

[3] Burke JP (2003). Infection control – a problem for patient safety. N Engl J Med.

[4] Gerberding JL (2003). Hospital-onset infections: a patient safety issue. Ann Intern Med.

[5] Jeeva RR, Wright D (2014). Healthcare-associated infection: a national patient safety problem and the coordinated response. Med Care.

[6] Krein SL, Olmsted RN, Hofer TP, Kowalski C, Forman J, Banaszak-Holl J, Saint S (2006). Translating infection prevention evidence into practice using quantitative and qualitative research. Am J Infect Control.

[7] Saint S, Kowalski CP, Banaszak-Holl J, Forman J, Damschroder L, Krein SL (2009). How active resistors and organizational conspirators affect healthcare-acquired infection prevention efforts. Jt Comm J Qual Patient Saf.

[8] Health Protection Agency (Now Public Health England) (2013). Quarterly Epidemiological Commentary: Mandatory MRSA, MSSA and *E. Coli* bacteraemia and *C. difficile* infection data.

[9] Health Protection Scotland (2012). Quarterly report on the surveillance of *Clostridium difficile* infection (CDI) in Scotland.

[10] HSC Northern Ireland (2012). Public Health Agency. *C. difficile* Surveillance quarterly report.

[11] Public Health Wales (2012). *Clostridium difficile* mandatory surveillance report: All Wales.

[12] Department of Health. UK Five Year Antimicrobial Resistance Strategy 2013 to 2018. London: Department of Health; 2013.

[13] Welsh Government. Together for Health: Tackling antimicrobial resistance and improving antibiotic prescribing. A Delivery Plan for Wales NHS and its partners. Cardiff: Welsh Government; 2016.

[14] Welsh Government (2014). Code of practice for the prevention and control of healthcare associated infections.

[15] Welsh Government (2011). Commitment to purpose: eliminating preventable healthcare associated infections (HCAIs).

[16] Dodds A, Kodate N (2011). Accountability, organisational learning and risks to patient safety in England: conflict or compromise. Health Risk Soc.

[17] Department of Health. An organisation with a memory. London: Department of Health; 2000.

[18] Leape LL, Berwick DM (2000). Safe health care: are we up to it?. BMJ.

[19] Vincent C, Taylor-Adams S, Chapman EJ, Hewett D, Prior S, Strange P, Tizzard A (2000). How to investigate and analyse clinical incidents: clinical risk management unit and association of litigation and risk management protocol. BMJ.

[20] Clarke S (1999). Perceptions of organisational safety: implications for the development of safety culture. J Organ Behav.

[21] De Bono S, Heling G, Borg MA (2014). Organisational culture and its implications for infection prevention and control in healthcare institutions. J Hosp Infect..

[22] Charani S, Holmes AH (2013). Antimicrobial stewardship programmes: the need for wider engagement’. BMJ Qual Saf.

[23] Conroy LJ, Raveis VH, Pogorzelska-Maziarz M, Uchida M, Stone PW, Larson EL (2013). Tensions inherent in the evolving role of the infection preventionist. Am J Infect Control.

[24] Brewster L, Tarrant C, Dixon-Woods M (2016). Qualitative study of views and experiences of performance management for healthcare associated infections. J Hosp Infect..

[25] Gould DJ, Hale R, Waters E, Allen D (2016). Promoting health workers’ ownership of infection prevention and control: using normalization process theory as an interpretive framework. J Hosp Infect..

[26] Lo E, Nicolle LE, Coffin SE, Gould C, Maragakis LL, Meddings J, Pegues DA, Pettis AM, Saint S, Yokoe DS (2014). Strategies to prevent catheter associated urinary tract infections in acute hospitals. Infect Control Hosp Epidemiol.

[27] Hale R, Powell T, Drey NS, Gould DJ (2015). Working practices and success of infection prevention and control teams: a scoping study. J Hosp Infect..

[28] Zimmerman B, Reason P, Rykert L, Gitterman L, Christian J, Gardam M (2013). Frontline ownership: generating a cure mindset for patient safety. Healthcare Papers.

[29] Masterton RG, Mifsud AJ, Rao GG (2003). Review of hospital isolation and infection control precautions. J Hosp Infect.

[30] Coia JE, Duckworth GJ, Edwards DI, Farrington M, Fry C, Humphreys H, Mallaghan C, Tucker DR (2006). Hospital infection society and Infection Control Nurses Association guidelines for the control of prevention of methicillin-resistant Staphylococcus aureus (MRSA) in healthcare facilities. J Hosp Infect..

[31] Cookson BD, Macrae MB, Barrett SP, Brown DF, Chadwick C, French GL, Hateley P, Hosein IK, Wade JJ (2006). Guidelines for the control of glycopeptides-resistant enterococci in hospitals. J Hosp Infect..

[32] Muto CA, Jernigan JA, Ostrowsky BE, Richet HM, Jarvis WR, Boyce JM, Farr BM (2003). SHEA guideline for preventing nosocomial transmission of multi-drug resistant strains of Staphylococcus aureus and enterococcus. Infect Control Hosp Epidemiol.

[33] Siegel JD, Rhinehart E, Jackson M, Chiarello L (2007). Guideline for isolation precautions: preventing transmission of infectious agents in healthcare settings. Am J Infect Control.

[34] Jackson MM, Lynch P (1996). Invited commentary: guideline for isolation precautions in hospitals. Am J Infect Control.

[35] Prieto J, Macleod Clark J (2005). Contact precautions for Clostridium difficile and methicillin-resistant Staphylococcus aureus: assessing the impact of a supportive intervention to improve practice. J Res Nurs.

[36] Cooper BS, Stone SP, Kibbler CC, Cookson BD, Roberts JA, Medley GF, Duckworth GJ, Lai R, Ebrahim S (2003). Systematic review of isolation policies in the hospital management of methicillin-resistant Staphylococcus aureus: a review of the literature with epidemiological and economic modelling. Health Technol Assess.

[37] Aboelela SW, Saiman L, Stone P, Lowy FD, Quiros D, Larson E (2006). Effectiveness of barrier precautions and surveillance cultures to control transmission of multidrug-resistant organisms: a systematic review of the literature. Am J Infect Control.

[38] Davies H, Rees J (2000). Psychological effects of isolation nursing (1): mood disturbance. Nurs Stand.

[39] Gammon, J. & Hunt, J. The stigmatisation of source isolation: a literature review. J Res Nurs In Press. 10.1177/1744987119845031 PMC7932329 34394593

[40] Gammon J, Hunt J (2018). Source isolation and patient wellbeing in healthcare settings: a literature review. Br J Nurs.

[41] Gammon J, Hunt J (2018). A review of isolation practices and procedures in healthcare settings. Br J Nurs.

[42] Knowles H (1993). The experience of infectious patients in isolation. Nurs Times.

[43] Rees J, Davies H, Birchall C (2000). Psychological effects of source isolation (2): patient satisfaction. Nurs Stand.

[44] Allaire Y, Firsirotu ME (1984). Theories of organizational culture. Organ Stud.

[45] Ashkanasy NM, Jackson CRA, Anderson N, Ones DS, Sinangil HK, Viswesvaran C (2001). Organizational culture and climate. Handbook of industrial work and organizational psychology. Volume 2: organizational psychology.

[46] Alvesson M (1995). Cultural perspectives on Organisations.

[47] Ott JS (1989). The organizational culture perspective.

[48] Brown A (1995). Organizational Culture.

[49] Hunt J, Sanchez A, Tadd W, O’Mahony S (2012). Organizational culture and performance in health care for older people: a systematic review. Rev Clin Gerontol.

[50] Schein EH (1985). Organisational culture and leadership.

[51] Mannion R, Davies HTO, Marshall MN (2005). Cultures for performance in health care.

[52] Scott JT, Mannion R, Davies HTO, Marshall MN (2001). Organisational culture and performance in the NHS: a review of the theory, instruments and evidence.

[53] Scott JT, Mannion R, Davies HTO, Marshall MN (2003). Health care performance and Organisational culture.

[54] Scott JT, Mannion R, Davies HTO, Marshall MN (2003). Does organisational culture influence health care performance? A review of the evidence. J Health Serv Res Policy.

[55] Scott JT, Mannion R, Davies HTO, Marshall MN (2003). Implementing culture change in health care: theory and practice. Int J Qual Health Care.

[56] May C (2013). Agency and implementation: understanding the embedding of healthcare innovations in practice. Soc Sci Med.

[57] Colyvas JA, Jonsson S (2011). Ubiquity and legitimacy: disentangling confusion and institutionalization. Sociol Theor.

[58] May C (2006). A rational model for assessing and evaluating complex interventions in health care. BMC Health Serv Res.

[59] May C (2013). Towards a general theory of implementation. Implement Sci.

[60] Coffey A, Atkinson P (1996). Making sense of qualitative data: complementary research strategies.

[61] Ayres L, Kavanaugh K, Knafl KA (2003). Within-case and across-case approaches to qualitative data analysis. Qual Health Res.

