# Supplementary material for: Infection prevention control and organisational patient safety culture within the context of isolation: study protocol
Source: BMC Health Serv Res. 2019 May 8;19:296. doi: 10.1186/s12913-019-4126-x (PMC6507018; doi:10.1186/s12913-019-4126-x)
Supplement: Supplementary file 1 — Interview Topic Guide - Patient. (DOCX 18 kb) [file 12913_2019_4126_MOESM1_ESM.docx]

**Infection Prevention Control and Organisational Patient Safety Culture within the Context of Isolation**

**Interview Topic Guide: Patient**

**Introduction**

Hello - Thank you for agreeing to meet with us. My name is (………) and I am a researcher on the Infection Prevention Control and Organisational Patient Safety Culture within the Context of Isolation study, in which you agreed to take part.

Today I would like us to talk about your experiences in hospital and how this made you feel.

This discussion is in strict confidence and nothing that you say today will identify you with our research. I really want to look at improving situations and therefore I am interested in your personal concerns and the experiences you have had. There are no right or wrong answers and if you have any worries or concerns, then just stop and ask me. With your consent, the discussion will be recorded but again, everything is in strict confidence.

**1: Tell me about your most recent hospital experience as an inpatient in isolation.**

**2: What were the excellent experiences?**

**3: What were the negative experiences?**

**4: What factors led to the excellent and negative experiences?**

What was it that made those experiences excellent and negative?

**5: Were you involved in decisions about the medical treatment and personal care you received in hospital and your moving to an isolation room?**

Were you involved in planning your discharge from isolation?

**6: Do you know why you were placed in isolation?**

**7: Was personal choice important to you?**

**8: Was your stay in isolation / hospital as you expected?**

**9: How did staff treat you during your stay in hospital isolation?**

What are your views regarding the staff that cared for you?

**10: Were there times you were upset by something you saw or heard on the ward, relating to IPC and / or patient safety?**

**11: Were there times when you were impressed by something you saw or heard on the ward, relating to IPC and / or patient safety?**

**12: Did you feel safe and secure during your stay in hospital?**

What was it that made you feel safe and secure?

What were the reasons for you not feeling safe and secure?

**13: In what ways do you understand IPC and what IPC involves?**

Are there any examples you can think of where you witnessed good IPC?

Are there any examples you can think of where you witnessed poor IPC?

**14: In what ways do you understand patient safety culture?**

What would positive patient safety culture look like to you?

What would poor patient safety culture look like to you?

**15: Overall, how would you rate IPC and patient safety on the ward in which you were placed in hospital isolation?**

**16: How should we measure the quality of patent safety care people like you receive during your stay in hospital?**

IE: Good care / bad care.

**And finally:**

**17: What did it mean to you to be placed in isolation?**

What was most difficult about your stay in isolation?

Are there any ways you can think of that the experience of being placed in isolation can be made more positive for patients?
