# Supplementary material for: Infection prevention control and organisational patient safety culture within the context of isolation: study protocol
Source: BMC Health Serv Res. 2019 May 8;19:296. doi: 10.1186/s12913-019-4126-x (PMC6507018; doi:10.1186/s12913-019-4126-x)
Supplement: Supplementary file 2 — Interview Topic Guide - Relative / Informal Carer. (DOCX 18 kb) [file 12913_2019_4126_MOESM2_ESM.docx]

**Infection Prevention Control and Organisational Patient Safety Culture within the Context of Isolation**

**Interview Topic Guide: Relative / Informal Carer**

**Introduction**

Hello - Thank you for agreeing to meet with us. My name is (………) and I am a researcher on the Infection Prevention Control and Organisational Patient Safety Culture within the Context of Isolation study, in which you agreed to take part.

Today I would like us to talk about your relative’s experiences in hospital and how this made you feel.

This discussion is in strict confidence and nothing that you say today will identify you with our research. I really want to look at improving situations and therefore I am interested in your personal concerns and the experiences you have had. There are no right or wrong answers and if you have any worries or concerns, then just stop and ask me. With your consent, the discussion will be recorded but again, everything is in strict confidence.

**1: Tell me about your relative’s / friend’s most recent hospital experience as an inpatient in isolation.**

**2: What were the excellent experiences?**

**3: What were the negative experiences?**

**4: What factors led to the excellent and negative experiences?**

What was it that made those experiences excellent and negative?

**5: Was your relative / friend (and you) involved in decisions about the medical treatment and personal care they received in hospital and their moving to an isolation room?**

Was your relative / friend (and you) involved in planning their discharge from isolation?

**6: Do you know why they were placed in isolation?**

**7: Was personal choice important to you?**

**8: Was their stay in isolation / hospital as you expected?**

**9: How did staff treat your relative (and you) during their stay in isolation in hospital?**

What are your views regarding the staff that cared for your relative / friend?

**10: Were there times you were upset by something you saw or heard on the ward, relating to IPC and / or patient safety?**

**11: Were there times when you were impressed by something you saw or heard on the ward, relating to IPC and / or patient safety?**

**12: Do you feel your relative / friend was safe and secure during their stay in hospital?**

What was it that made you feel they were safe and secure?

What were the reasons for you not feeling they were safe and secure?

**13: In what ways do you understand IPC and what IPC involves?**

Are there any examples you can think of where you witnessed good IPC?

Are there any examples you can think of where you witnessed poor IPC?

**14: In what ways do you understand patient safety culture?**

What would positive patient safety culture look like to you?

What would poor patient safety culture look like to you?

**15: Overall, how would you rate the quality of the care your relative / friend received in hospital isolation?**

**16: How should we measure the quality of patent safety care people like your relative / friend receive during their stay in hospital?**

IE: Good care / bad care.

**And finally:**

**17: What did it mean to you for your relative / friend to be placed in isolation?**

What was most difficult about their stay in isolation?

Are there any ways you can think of that the experience of being placed in isolation can be made more positive for patients?
