# Supplementary material for: Infection prevention control and organisational patient safety culture within the context of isolation: study protocol
Source: BMC Health Serv Res. 2019 May 8;19:296. doi: 10.1186/s12913-019-4126-x (PMC6507018; doi:10.1186/s12913-019-4126-x)
Supplement: Supplementary file 3 — Interview Topic Guide - Key Staff. (DOCX 18 kb) [file 12913_2019_4126_MOESM3_ESM.docx]

**Infection Prevention Control and Organisational Patient Safety Culture within the Context of Isolation**

**Interview Topic Guide: Key Staff**

**Introduction**

Hello - Thank you for agreeing to meet with us. My name is (………) and I am a researcher on the Infection Prevention Control and Organisational Patient Safety Culture within the Context of Isolation study, in which you agreed to take part.

This discussion is in strict confidence and nothing that you say today will identify you with our research. I really want to look at improving situations and therefore I am interested in your personal concerns and the experiences you have had. There are no right or wrong answers and if you have any worries or concerns, then just stop and ask me. With your consent, the discussion will be recorded but again, everything is in strict confidence.

**1: Tell me about your experience working within the NHS.**

**2: How long have you been working in this particular organisation?**

**3: What are your main roles within the organisation?**

**4: In your experience, what organisational factors lead to positive and negative IPC policy and procedures?**

**5: What patient safety measures do you currently have in place?**

What are they used for?

How are they used?

What are your quality measures?

**6: Does IPC and patient safety feature at Health Board meetings?**

In what ways does it feature?

In what ways is feedback from patients fed to the Health Board?

**7: In what ways is information from frontline staff fed through to the Health Board?**

**8: How does the Health Board demonstrate the ways in which staff are encouraged to take responsibility for IPC and patient safety within the organisation?**

**9: How does the Health Board deal with staff who may not measure-up to expectations, for whatever reason?**

IE: Illness, attitude, drug errors, non-compliance with IPC and patient safety policy and procedure.

**10: How are adverse incidents identified and managed?**

What would be included as an adverse incident?

IE: Falls, drug errors, non-compliance with IPC and patient safety policy and procedure, IPC and patient safety procedure related deaths.

**11: In what ways are patients offered choice?**

IE: In relation to medical care and treatment in isolation, social and personal aspects of isolation.

**12: Are patients informed when things go wrong?**

In what ways are they informed?

**13: What is the Health Board’s strategy for supporting, including social and emotional support, for patients in isolation?**

IE: Stigma, loneliness, reduced contact with other patients, limited visiting, less interaction with hospital staff.

**And finally:**

**14: In what ways do you understand IPC ownership?**

What does it mean to you?

Are there any examples you have witnessed of staff IPC ownership?

**15: In what ways do you understand patient safety culture?**

What would positive patient safety culture look like, to you?

What would poor patient safety culture look like to you?
