# Supplementary material for: Infection prevention control and organisational patient safety culture within the context of isolation: study protocol
Source: BMC Health Serv Res. 2019 May 8;19:296. doi: 10.1186/s12913-019-4126-x (PMC6507018; doi:10.1186/s12913-019-4126-x)
Supplement: Supplementary file 4 — Interview Topic Guide - Ward Staff. (DOCX 19 kb) [file 12913_2019_4126_MOESM4_ESM.docx]

**Infection Prevention Control and Organisational Patient Safety Culture within the Context of Isolation**

**Interview Topic Guide: Ward Staff**

**Introduction**

Hello - Thank you for agreeing to meet with us. My name is (………) and I am a researcher on the Infection Prevention Control and Organisational Patient Safety Culture within the Context of Isolation study, in which you agreed to take part.

This discussion is in strict confidence and nothing that you say today will identify you with our research. I really want to look at improving situations and therefore I am interested in your personal concerns and the experiences you have had. There are no right or wrong answers and if you have any worries or concerns, then just stop and ask me. With your consent, the discussion will be recorded but again, everything is in strict confidence.

**1: Tell me about your experience working within the NHS.**

**2: How long have you been working on this ward?**

**3: What does your role involve?**

**4: What preparation have you had for this role?**

**5: What do you enjoy most about your work?**

**6: What do you enjoy least about your work?**

**7: In your experience, what organisational factors lead to positive and negative IPC policy and procedures?**

**8: In what ways are you encouraged to take responsibility for IPC on the ward and within the organisation?**

Training

Education

**9: What patient safety measures are in place, on the ward?**

What are they used for?

How are they used?

What are your quality measures?

**10: How do you know when / if you are doing well?**

**11: Do you have regular appraisals?**

**12: If a staff member is not performing well, how is this dealt with on the ward and within the Health Board?**

**13: Do you feel valued by the Health Board?**

**14: Are your concerns listened to by senior management?**

What is the outcome of this?

**15: By what criteria would you judge a ward to be good or bad, regarding IPC and patient safety?**

**16: How are adverse incidents recorded and dealt with?**

What would be included as an adverse incident?

IE: Falls, drug errors, non-compliance with IPC and patient safety policy and procedure, IPC and patient safety procedure related deaths.

**17: In what ways are patients informed when things go wrong?**

**18: In what ways are patients offered choice?**

IE: In relation to medical care and treatment in isolation, social and personal aspects of isolation.

**19: What patients do you see as being the most challenging in terms IPC and isolation procedures, in terms of delivering quality of care?**

Why are they challenging?

In what ways are they challenging?

**20: For patients in isolation, the experience can sometimes lead to feelings of loneliness, social isolation and stigmatisation. What services / resources are available to you to ensure patients are provided with support, including social and emotional support, to prevent the adverse effects of the restrictions of barrier nursing, as much as possible?**

**21: What helps you deliver safe and effective IPC and patient safety care?**

**22: What prevents you from delivering IPC and patient safety care?**

**And finally:**

**23: In what ways do you understand IPC ownership?**

What does it mean to you?

Are there any examples you can think of regarding IPC ownership – Either experienced yourself or witnessed in others?

**24: In what ways do you understand patient safety culture?**

What would positive patient safety culture look like, to you?

What would poor patient safety culture look like to you?
